# Artificial intelligence in urology: Revolutionizing diagnostics and treatment planning

**DOI:** 10.1080/20905998.2024.2443334

**Published:** 2024-12-19

**Authors:** Kirolos Eskandar

**Affiliations:** General Medicine, Diakonie Klinik, Mosbach, Germany

**Keywords:** Artificial intelligence, urology, diagnostics, personalized medicine, robotic surgery

## Abstract

Artificial Intelligence (AI) is rapidly transforming the field of urology, offering unprecedented advancements in diagnostics and treatment planning. This review explores the integration of AI across various urological practices, highlighting its impact on improving diagnostic accuracy, optimizing treatment strategies, and enhancing patient monitoring. We examine the role of AI in imaging, pathology, and personalized medicine, as well as its contributions to robotic-assisted surgeries and remote patient care. The article also addresses the ethical and legal challenges posed by AI, including issues of data privacy, algorithmic bias, and regulatory oversight. Despite these challenges, the potential of AI to revolutionize urology is immense, promising more precise, efficient, and patient-centered care. As AI technologies continue to evolve, their integration into urology will likely lead to significant improvements in patient outcomes and the overall quality of care.

## Introduction

Artificial intelligence (AI) has rapidly evolved from a theoretical concept to a transformative technology in medicine. Its adoption is particularly notable in fields like urology, where AI’s potential to enhance diagnostics and treatment planning is beginning to be realized. AI technologies encompass several key components, including machine learning (ML), deep learning, neural networks, and natural language processing (NLP). These technologies are designed to simulate human cognitive processes, enabling machines to learn from data, identify patterns, and make decisions with minimal human intervention.

Machine learning, a subset of AI, is central to these advancements. It involves algorithms that improve their performance on a task over time through experience. Deep learning, a more specialized branch of ML, uses layered neural networks to analyze various forms of data, such as medical images and patient records. These networks mimic the human brain’s structure, allowing for more complex analyses and predictions. NLP, another critical AI technology, focuses on enabling machines to understand and respond to human language. In medicine, NLP is particularly useful for analyzing unstructured data like clinical notes, which can then be used to improve patient outcomes and streamline clinical workflows [1, 2].

Historically, AI’s role in healthcare has been relatively modest, limited to early diagnostic tools and data management systems. However, over the past two decades, significant advances in computational power and data availability have accelerated AI’s integration into medical practice. In urology, AI is being harnessed for a variety of applications, from predicting patient outcomes to optimizing surgical procedures. The increasing availability of big data, combined with advances in AI algorithms, has made it possible to develop predictive models that can assist in diagnosing conditions like prostate cancer and renal diseases with unprecedented accuracy [1].

The adoption of AI in urology is part of a broader trend in medicine, where AI-driven tools are increasingly being used to augment clinical decision-making. As AI technologies continue to evolve, their applications in urology are expected to expand, offering new opportunities for personalized medicine and improved patient care [2].

### Methodology

#### Search strategy

A systematic literature review was conducted following the PRISMA (Preferred Reporting Items for Systematic Reviews and Meta-Analyses) guidelines to ensure a transparent and replicable process. A comprehensive search was performed across multiple reputable databases, including PubMed, Google Scholar, Scopus, and Web of Science. The search was conducted between 1 January 2024, and 30 June 2024. The search strategy was meticulously developed to ensure precision and comprehensiveness.

The following keywords and Medical Subject Headings (MeSH) were used in the search query: ‘Artificial Intelligence,’ ‘Urology,’ ‘Diagnostics,’ ‘Personalized Medicine,’ ‘Robotic Surgery,’ ‘Machine Learning,’ and ‘Deep Learning.’ Boolean operators (AND, OR) were used to combine these terms and refine the search results. The search was limited to peer-reviewed articles published in English.

#### Study selection

The inclusion criteria for selecting studies were as follows:

- Language: Articles published in English.
- Focus: Studies explicitly addressing the use of AI in urology, including its applications in diagnostics, treatment planning, personalized medicine, and robotic surgery.
- Study Type: Original research articles, systematic reviews, and meta-analyses.
- Publication Date: Studies published between 2018 – 2024.

Exclusion criteria included:

1. Non-peer-reviewed sources: Articles such as editorials, commentaries, and conference abstracts were excluded.
2. Non-relevant Focus: Studies that did not focus directly on AI applications in urology were excluded.

#### Screening process

The initial search yielded 139 articles. Duplicate records were removed using reference management software (e.g. EndNote or Mendeley). The remaining articles were subjected to a two-stage screening process:

1. Title and Abstract Screening: The titles and abstracts of the remaining articles were carefully reviewed to assess their relevance to the topic. Articles that did not meet the inclusion criteria were excluded at this stage.
2. Full-Text Screening: Articles that passed the initial screening were then assessed for eligibility through a full-text review. Each article was meticulously evaluated against the inclusion and exclusion criteria. Any ambiguities or uncertainties encountered during this process were resolved by the author through a detailed examination of the study’s methodology and findings.

Out of the 139 articles initially identified, 26 met all inclusion criteria and were included in the final review (Table 1).

**Table 1.** Summary of articles reviewed, highlighting study focus, AI techniques, and key findings.

Reference	Study Focus/Area	AI Techniques Used	Key Findings/Implications
[1]	AI in robotics and automation	Machine learning, deep learning	Highlighted AI’s role in advanced robotics, offering improved precision and adaptability.
[2]	Overview of deep learning techniques	Deep learning frameworks	Provided taxonomy of deep learning, emphasizing future applications in healthcare.
[3]	AI in prostate cancer detection	Radiomics, ML	Validated AI’s diagnostic performance on MRI, proving non-inferiority to radiologists.
[4]	AI in urological diagnostics and treatment	Various AI tools	Discussed AI’s potential in personalized urology care and early diagnosis.
[5]	Gleason grading in prostate cancer	Deep learning	Demonstrated high accuracy in histopathologic diagnosis using DL models.
[6]	AI in bladder cancer diagnosis	AI algorithms	Reviewed advancements and emphasized future AI roles in early cancer detection.
[7]	Impact of AI on urological diseases	Comprehensive review	Highlighted AI’s transformative potential in urology and associated challenges.
[8]	Predictive cancer patient digital twins	Predictive analytics, AI simulations	Proposed AI-driven digital twins for personalized oncology care.
[9]	AI-assisted pathology in prostate cancer	Microsimulation, cost-effectiveness models	Showed cost-effectiveness and diagnostic improvements in prostate cancer.
[10]	Evolution of robotic surgery in prostate cancer	Robotic AI systems	Chronicled 20 years of AI integration into robotic-assisted surgeries.
[11]	Remote monitoring via wearable sensors	Sensor analytics, AI	Discussed AI’s role in patient monitoring, especially in critical care.
[12]	AI-based wearable sensors	Machine learning, IoT	Explored wearable sensors’ applications in digital health and diagnostics.
[13]	AI in remote patient monitoring	AI models for monitoring	Emphasized AI’s integration into patient care and operational efficiency.
[14]	Post-COVID telehealth developments	AI-driven telehealth	Highlighted the rise of telehealth as a mainstream healthcare solution.
[15]	Ethics in AI for mental health	Ethical AI frameworks	Explored responsible AI implementation in mental health interventions.
[16]	AI perception in healthcare	AI adoption trends	Addressed misconceptions and realities of AI in healthcare systems.
[17]	Bias in machine learning	Ethical considerations	Analyzed implications of algorithmic bias in clinical AI applications.
[18]	Deep learning in healthcare	Deep learning	Provided a comprehensive guide to DL applications and potential in medicine.
[19]	AI bias and safety in healthcare	Ethical AI	Highlighted risks of AI bias and strategies for clinical safety.
[20]	AI in liability and tort law	Legal frameworks	Discussed legal implications of AI-caused injuries in clinical settings.
[21]	Algorithmic bias in health systems	Ethical AI, ML	Examined systemic risks and mitigation strategies in AI deployments.
[22]	AI in health care	Strategic AI applications	Explored promises and pitfalls of AI in transforming healthcare.
[23]	AI in urological cancers	AI-based diagnostics	Discussed current and future trends of AI in urological oncology.
[24]	AI in genome editing	AI-enhanced gene editing	Reviewed AI’s applications and challenges in advancing genome editing.
[25]	Precision and genomic medicine	AI in genomics	Focused on AI’s role in precision medicine for improved outcomes.
[26]	AI in precision oncology	AI algorithms, ML	Emphasized the integration of AI into precision oncology for tailored treatments.

#### Data extraction

Data from the selected studies were extracted using a standardized form. The extracted data included:

- Study Characteristics: Authors, year of publication, country, study design, and sample size.
- AI Application: Specific AI technology used (e.g. machine learning algorithms, deep learning models), area of application in urology (e.g. diagnostics, treatment planning), and reported outcomes.
- Study Outcomes: Primary and secondary outcomes reported in the studies, including diagnostic accuracy, treatment efficacy, and any reported limitations or biases.

The data extraction process was independently conducted by two reviewers, with discrepancies resolved through discussion.

#### Quality assessment

To assess the quality of the included studies, the Joanna Briggs Institute (JBI) Critical Appraisal Tools were utilized. These tools provide a structured approach to evaluating the methodological quality of studies, considering factors such as study design, risk of bias, and validity of results. Each study was rated as high, moderate, or low quality. Only studies rated as high or moderate quality were included in the synthesis.

#### Data synthesis

The findings from the included studies were synthesized using a narrative approach, organized by the specific applications of AI in urology. Key themes identified across the studies included diagnostic accuracy, the role of AI in personalized treatment planning, and the integration of AI with robotic surgery. Where possible, quantitative data were summarized using descriptive statistics. Heterogeneity in study design and outcomes precluded meta-analysis.

**Figure 1.**
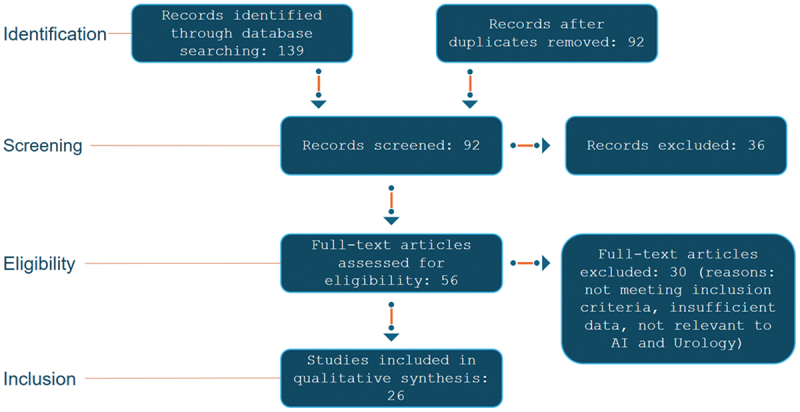
Illustrates the PRIMSA flow diagram.

To provide a transparent overview of the study selection process, a PRISMA flow diagram is included (Figure 1), detailing the number of records identified, screened, and included in the review, along with reasons for exclusion at each stage.

## AI in urologic diagnostics

Artificial Intelligence (AI) is significantly advancing urologic diagnostics by improving the accuracy, efficiency, and personalization of diagnostic procedures. The integration of AI into imaging, pathology, and laboratory testing in urology is transforming the landscape, particularly in the detection and characterization of urologic cancers.

In imaging, AI has made substantial strides, particularly in the diagnosis of prostate cancer using multiparametric MRI (mpMRI). Prostate cancer diagnosis has traditionally relied heavily on the expertise of radiologists, who interpret mpMRI images to detect abnormalities. However, this process is subject to human error, including variability in interpretation. AI-assisted MRI interpretation has emerged as a game-changer, enhancing both the sensitivity and specificity of prostate cancer detection. AI algorithms, trained on large datasets of mpMRI scans, are capable of identifying patterns and features that may be indicative of prostate cancer with greater precision than traditional methods. These AI systems can highlight suspicious areas within the prostate, guide biopsies, and help in the early detection of clinically significant prostate cancers, which might otherwise be missed by the human eye [3].

For renal masses, AI is similarly transformative. Renal masses can be benign or malignant, and accurate differentiation is critical for determining the appropriate course of treatment. Traditionally, radiologists have used CT scans to analyze these masses, but distinguishing between benign and malignant masses can be challenging, particularly in complex cases. AI-driven CT scan analysis provides an advanced tool for radiologists, improving the diagnostic accuracy by analyzing the radiomic features of renal masses. These AI models, trained on thousands of CT images, can assess characteristics such as texture, shape, and vascularity, which are critical in differentiating between benign and malignant lesions. This enhances the diagnostic process, leading to better-informed clinical decisions and potentially reducing unnecessary surgeries [4].

In the field of pathology, AI is making histopathological analysis more accurate and consistent. Prostate biopsy evaluation, a critical step in diagnosing prostate cancer, has historically been prone to variability due to differences in pathologist interpretations. AI tools are now being employed to standardize this process. These tools analyze biopsy samples using deep learning algorithms that can detect subtle histological patterns indicative of cancer. For example, AI can assist in grading prostate cancer by identifying the architectural patterns associated with different Gleason scores, reducing inter-observer variability, and ensuring a more consistent and objective diagnosis [5]. Furthermore, AI-based predictive models are being developed to forecast tumor aggressiveness and potential progression. By integrating genomic and clinical data, these models help in stratifying patients based on risk, which is crucial for personalized treatment planning and management.

In laboratory testing, AI has significantly improved the accuracy of urine cytology, particularly in the detection of bladder cancer. Traditional urine cytology, while useful, has limitations in sensitivity, especially in detecting low-grade tumors. AI algorithms have been developed to analyze cytology samples more precisely, identifying cancerous cells with higher accuracy. These AI tools can detect subtle cellular changes that might be overlooked in manual examinations, thus enhancing early detection and potentially reducing the need for more invasive diagnostic procedures like cystoscopy [6]. Additionally, AI is at the forefront of biomarker discovery in urology. By processing large-scale omics data, AI helps identify novel biomarkers that can be used for the early detection of urological diseases, prognostication, and monitoring response to therapy. This approach is particularly promising in the development of non-invasive tests, which could revolutionize the diagnosis and management of conditions such as prostate and bladder cancer [7].

## AI in urologic treatment planning

The application of artificial intelligence (AI) in urologic treatment planning is transforming the landscape of personalized medicine, particularly in prostate cancer management. AI-driven models are now increasingly employed to stratify risk and optimize treatment decisions for patients with prostate cancer. These AI models analyze a plethora of data, including genomic, imaging, and clinical information, to predict the aggressiveness of tumors and recommend personalized treatment strategies. For instance, AI can refine the accuracy of risk stratification by integrating data from multiple sources, which enables clinicians to make more informed decisions about whether to pursue active surveillance, surgery, or other interventions. This level of precision is crucial in avoiding overtreatment or undertreatment, thus improving patient outcomes [8].

In the realm of robotic surgery, AI is enhancing both the precision and safety of procedures. Robotic-assisted surgery, particularly in prostate cancer, has evolved significantly with the integration of AI technologies. These advancements have made surgeries more precise, reducing the likelihood of complications and improving functional outcomes post-surgery. AI-enhanced robotics systems now assist surgeons by providing real-time data and predictive analytics, which guide decision-making during the procedure. Moreover, these systems are becoming increasingly sophisticated, incorporating machine learning algorithms that continually improve based on new data, thereby enhancing their effectiveness over time [9].

AI is also playing a pivotal role in predicting surgical outcomes. Predictive models powered by AI are now capable of estimating postoperative complications and long-term outcomes with greater accuracy than traditional methods. These models analyze historical data, including patient demographics, tumor characteristics, and surgical details, to forecast potential risks and benefits of various treatment options. This predictive capability is invaluable in pre-surgical planning, as it allows for the customization of treatment plans that are tailored to the individual patient’s risk profile, ultimately improving both the safety and efficacy of urologic surgeries [8].

## AI in urologic patient monitoring and follow-up

Artificial Intelligence (AI) is transforming patient monitoring and follow-up in urology by enhancing remote monitoring and predictive analytics, ensuring more personalized and proactive patient care. AI’s integration into telemedicine platforms and wearable devices is a significant advancement, enabling continuous and real-time monitoring of patients, particularly those with chronic urological conditions.

AI-powered teleurology platforms are revolutionizing remote monitoring by allowing continuous observation of patients’ health statuses. These platforms utilize machine learning algorithms to process vast amounts of data collected from various sources, including electronic health records and wearable devices [10]. This approach ensures that any changes in a patient’s condition are promptly detected, allowing for timely intervention. For instance, patients with prostate cancer or those undergoing post-operative care can be monitored continuously, reducing the need for frequent hospital visits and enabling early detection of complications, thus improving outcomes [11].

Wearable devices integrated with AI are particularly beneficial in urology, where they can monitor vital signs, physical activity, and even specific biochemical markers relevant to urological health. These devices, such as smartwatches or specialized biosensors, continuously collect and transmit data, which AI algorithms then analyze to provide actionable insights. For example, AI can analyze data from wearable sensors to detect early signs of bladder cancer recurrence or monitor renal function in patients with chronic kidney disease. The ability to track and analyze this data in real-time enhances personalized care and allows for early intervention, ultimately improving patient outcomes [12].

In predictive analytics, AI models are being developed to forecast the likelihood of disease recurrence in conditions like prostate and bladder cancer. By analyzing historical data and identifying patterns, these models can predict the risk of recurrence with high accuracy, allowing clinicians to tailor follow-up schedules and treatment plans according to individual patient risk profiles. This personalized approach not only improves the efficiency of follow-up care but also minimizes the psychological burden on patients by reducing unnecessary tests and procedures [13].

Moreover, AI is being used to design surveillance programs that adapt to the specific risk factors of each patient. These programs utilize AI to analyze patient data continuously and adjust follow-up intervals based on the patient’s evolving health status. For instance, in prostate cancer patients, AI can help determine the optimal timing for follow-up imaging or laboratory tests, balancing the need for vigilance with the goal of minimizing patient burden and healthcare costs [14].

## Ethical and legal considerations

The integration of artificial intelligence (AI) into urology raises several ethical and legal considerations that must be addressed to ensure its responsible and equitable use. One of the most pressing concerns is data privacy and security. AI systems often rely on vast amounts of sensitive patient data, which, if not properly managed, could lead to significant privacy breaches. The complexity of AI algorithms, combined with the centralized storage of patient information, increases the risk of cyberattacks and unauthorized access. Ensuring robust data encryption, secure data storage, and strict access controls are essential to protecting patient information [15, 16].

Bias and fairness in AI algorithms also present critical challenges. AI systems can unintentionally perpetuate or even exacerbate existing biases, leading to inequitable healthcare outcomes. For instance, if an AI model is trained predominantly on data from a specific demographic group, it may not perform as accurately for other populations. This issue of bias could lead to disparities in diagnosis, treatment planning, and overall patient care. Addressing this requires developing AI systems with diverse and representative data sets, along with ongoing monitoring and adjustment of algorithms to mitigate bias [17].

The regulatory landscape surrounding AI in urology is still evolving. Current regulations are often insufficient to fully address the unique challenges posed by AI technologies. As AI continues to advance, there is a growing need for more comprehensive and specific guidelines that cover the ethical development, deployment, and monitoring of AI systems in healthcare. This includes ensuring transparency in AI decision-making processes, obtaining informed consent from patients, and establishing accountability for AI-driven outcomes. Regulatory bodies are beginning to draft frameworks, but ongoing efforts are required to keep pace with the rapid advancements in AI technology [17].

## Challenges and limitations of AI in urology

The integration of artificial intelligence (AI) into urology holds immense potential but is not without its challenges and limitations. One of the primary concerns is the quality and availability of data used to train AI models. AI algorithms are highly dependent on large, diverse, and high-quality datasets to ensure accurate predictions and generalizability across different populations. However, in urology, data quality can vary significantly due to differences in imaging techniques, equipment, and the variability in clinical practices across institutions. This variability can lead to biases in AI models, potentially reducing their accuracy and effectiveness when applied in diverse clinical settings [18]. Furthermore, the issue of dataset shift – where the data distribution changes over time due to evolving clinical practices or population demographics – poses a significant challenge. AI models trained on outdated or non-representative data may fail to perform adequately in real-world clinical environments, necessitating continuous monitoring and retraining of these models to maintain their efficacy [19].

Another significant barrier to the widespread adoption of AI in urology is the integration of AI tools into existing clinical workflows. For AI to be effective, it must seamlessly fit into the day-to-day operations of healthcare providers without disrupting the standard care processes. However, many AI tools are not designed with the end-user in mind, leading to difficulties in implementation. For instance, the output of AI models is often not presented in a manner that is easily interpretable by clinicians, which can hinder its adoption. Clinicians may struggle to understand how the AI’s recommendations are derived, especially when these models operate as ‘black boxes’ without providing clear explanations for their decisions [20]. This lack of transparency can lead to skepticism and resistance among healthcare providers, who may be reluctant to rely on AI tools for critical clinical decisions.

The acceptance and trust of AI by both clinicians and patients are also crucial for its successful implementation. Many clinicians express concerns about the reliability and safety of AI tools, particularly when these tools are used for high-stakes decisions such as cancer diagnosis or treatment planning. There is also a fear that over-reliance on AI could lead to a loss of clinical skills among healthcare providers [21]. From the patient’s perspective, the use of AI in healthcare raises concerns about the dehumanization of care, where decisions are made by algorithms rather than by physicians who understand the nuances of individual patient cases [22]. Additionally, the medicolegal implications of AI in clinical practice are still evolving, with questions about liability in cases where AI recommendations lead to adverse outcomes remaining largely unresolved.

## Future directions and innovations

As artificial intelligence (AI) continues to evolve, its potential applications in urology are expanding rapidly, opening up new frontiers in patient care and research. One of the most promising areas is the development of emerging AI technologies that are poised to revolutionize urology. These include advancements in deep learning and natural language processing that could enable more sophisticated diagnostic tools and treatment algorithms. For instance, AI-driven models are becoming increasingly adept at interpreting complex imaging data, identifying patterns that might be missed by human clinicians, and offering predictive insights that could guide personalized treatment plans. These emerging technologies are expected to play a critical role in improving diagnostic accuracy and treatment outcomes in urology, particularly in conditions like prostate cancer and kidney disease [23].

In the realm of genomic and molecular urology, AI holds tremendous potential for analyzing genetic data to deliver personalized care. With the integration of AI into genomic medicine, clinicians could soon tailor treatments based on a patient’s unique genetic profile. This approach is particularly relevant in the management of urological cancers, where AI can help identify genetic mutations that drive tumor growth and suggest targeted therapies. AI’s ability to process vast amounts of genomic data and generate actionable insights could lead to more effective and individualized treatment strategies, enhancing the precision of urologic care [24, 25].

Looking ahead, the future of AI in urology will likely involve more collaborative efforts between AI systems and human clinicians. This collaborative AI, where human expertise and machine intelligence work in tandem, could significantly enhance decision-making processes in urology. For example, AI could assist surgeons in planning and executing complex procedures by providing real-time data analysis and decision support, ultimately improving surgical outcomes. Moreover, as AI technologies continue to develop, they may also facilitate more efficient and effective training for urologists, using simulation-based learning environments powered by AI [23,26].

## Conclusion

In conclusion, the integration of artificial intelligence (AI) into urology represents a transformative advancement, offering significant improvements in diagnostics, treatment planning, patient monitoring, and personalized medicine. As AI technologies continue to evolve, their applications in imaging, pathology, and genomics are poised to enhance the precision and efficacy of urologic care. However, the successful adoption of AI in clinical practice will require addressing challenges related to data quality, integration into clinical workflows, and ensuring the acceptance and trust of both clinicians and patients. As the field progresses, a collaborative approach combining AI’s computational power with human expertise holds the promise of revolutionizing urology, ultimately leading to better patient outcomes and more personalized care.

## Data Availability

“Data sharing not applicable to this article as no data-sets were generated or analyzed during the current study”
